# dsRNA silencing of an R2R3-MYB transcription factor affects flower cell shape in a *Dendrobium* hybrid

**DOI:** 10.1186/s12870-015-0577-3

**Published:** 2015-08-11

**Authors:** Su-Ee Lau, Trude Schwarzacher, Rofina Yasmin Othman, Jennifer Ann Harikrishna

**Affiliations:** Centre for Research in Biotechnology for Agriculture, University of Malaya, 50603 Kuala Lumpur, Malaysia; Department of Biology, University of Leicester, University Road, Leicester, LE1 7RH United Kingdom; Institute of Biological Sciences, Faculty of Science, University of Malaya, 50603 Kuala Lumpur, Malaysia

**Keywords:** Cell morphogenesis, RNA interference, RNA silencing, Biotechnology, Gene silencing, Epidermal cell

## Abstract

**Background:**

The *R2R3*-*MYB* genes regulate pigmentation and morphogenesis of flowers, including flower and cell shape, and therefore have importance in the development of new varieties of orchids. However, new variety development is limited by the long breeding time required in orchids. In this study, we identified a cDNA, *DhMYB1*, that is expressed during flower development in a hybrid orchid, *Dendrobium hybrida* (*Dendrobium* bobby messina X *Dendrobium* chao phraya) then used the direct application of dsRNA to observe the effect of gene silencing on flower phenotype and floral epidermal cell shape.

**Results:**

Flower bud development in the *Dendrobium* hybrid was characterised into seven stages and the time of meiosis was determined as between stages 3 to 5 when the bud is approximately half of the mature size. Scanning electron microscopy characterisation of adaxial epidermal cells of the flower perianth, showed that the petals and sepals each are divided into two distinct domains based on cell shape and size, while the labellum comprises seven domains. Thirty-two partial cDNA fragments representing *R2R3-MYB* gene sequences were isolated from *D. hybrida*. Phylogenetic analysis revealed that nine of the translated sequences were clustered with MYB sequences that are known to be involved in cell shape development and from these, DhMYB1 was selected for full length cDNA cloning and functional study. Direct application of a 430 bp dsRNA from the 3’ region of *DhMYB1* to emerging orchid flower buds reduced expression of *DhMYB1* RNA compared with untreated control. Scanning electron microscopy of adaxial epidermal cells within domain one of the labellum of flowers treated with *DhMYB1* dsRNA showed flattened epidermal cells whilst those of control flowers were conical.

**Conclusions:**

*DhMYB1* is expressed throughout flower bud development and is involved in the development of the conical cell shape of the epidermal cells of the *Dendrobium* hybrida flower labellum. The direct application of dsRNA changed the phenotype of floral cells, thus, this technique may have application in floriculture biotechnology.

**Electronic supplementary material:**

The online version of this article (doi:10.1186/s12870-015-0577-3) contains supplementary material, which is available to authorized users.

## Background

Orchids*,* belonging to the family *Orchidaceae,* one of the largest and most evolved families among flowering plants [1] have a wide variety of attractive shapes and colours, long flowering life and availability throughout the year. This contributes to the economic importance of the orchid industry in Malaysia, Singapore, Thailand, China, Netherlands, Hawaii and continental USA [2], but to meet consumer demands continuous novel flower colours and shapes are expected and very desirable to the horticultural industry. However development of new varieties of orchids with different colours and shapes is limited by the long breeding time required (usually three to four years for one generation). Given these limitations, the development of time and cost effective approaches for manipulation of orchid flower colour and shape is needed.

Factors that determine flower colour include secondary metabolites, pH value, metal ions, flavonoid co-pigments, environmental temperature and morphology of the epidermal cells [3, 4]. In this study, we focused on the morphology of the epidermal cells. Epidermal cells are the first point of contact with both biotic and abiotic environments. Several types of flower epidermal cell shape have been characterised, for instance, conical, flat or pointed [5–9]. Together with flower shape, cell shape influences temperature and light capture of plant surfaces, and thus can affect flower colour [6, 7, 10]. For example, it has been suggested that a conical cell shape could enhance light absorption by the pigments by increasing the amount of incident light that enters the epidermal cells [10–12].

Plant physiology, including cell shape, is genetically controlled. MYB transcription factors regulate plant development and secondary metabolism, light and hormone signalling, cell morphogenesis and defence and stress responses [13, 14]. Most of the plant MYB transcription factors belong to the R2R3 subfamily (also known as 2R-MYB) [15], which are similar to R2 and R3 repeats of the animal c-MYB proteins [16–18]. R2R3 MYB subfamily members have a MYB domain which is constituted by two imperfect repeats of approximately 50–53 amino acids. Each repeat possesses three regularly spaced tryptophan residues that encode three α-helices with the second and third helix forming a helix–turn–helix conformation to bind to the target DNA [17–20]. However, in some exceptions a phenylalanine residue replaces the first tryptophan in the R3 repeat [17]. The AtMYB R2R3-type MYB transcription factors have been categorised into 23 subgroups according to conserved amino-acid sequence motifs present at the carboxyterminal of the MYB domain [14, 17]. In this study, we focused on subgroup 9, that is represented by the genes, *AmMIXTA*, *AtMYB16* and *PhMYB1*, reported to control cell differentiation in *Antirrhinum majus*, *Arabidopsis thaliana* and *Petunia hybrida* respectively and to activate anthocyanin biosynthetic gene expression [5, 12]. Several of the R2R3 MYB subgroup 9 members have further been associated with cell shape development including: *AmMYBML1*-induced production of both trichomes and conical cells in floral tissues [21]; AmMYBML2 extending the growth of conical petal epidermal cells [12]; AmMYBML3 enhancing cellular out-growth from the epidermis of all aerial organs [18] and the MIXTA-related R2R3 MYB gene of *Thalictrum thalictroides* promoting conical cell growth in the petal and carpel epidermal cells [22].

A number of approaches have been used for the improvement of flower quality and quantity with many successful examples using gene silencing (RNAi) in plants [23]. While RNAi modification has been widely applied *via* transgenic [23] and virus-based (VIGS) methods [9], agroinfiltration [24] and the direct mechanical inoculation of dsRNA [25, 26], have also been successful in inducing gene silencing. The direct application of dsRNA is attractive as a relatively rapid and low cost method compared to plant transformation, therefore, this approach was selected here to study the effect of silencing a MYB transcription factor during the development of orchid flowers. The aims of this study were first to describe the flower development and epidermal cell shape and patterns in *D. hybrida*, to identify MYB gene sequences involved in cell shape development of *D. hybrida* and thirdly to develop a simple and rapid approach to modify orchid flower cell shape using *D. hybrida* as a study model. We first characterised flower bud development in the *Dendrobium* hybrid into seven stages then used scanning electron microscopy to determine adaxial epidermal cell patterns in the flower perianth. Partial R2R3-MYB gene sequences were isolated from *D. hybrida* and were used in phylogenetic analysis to identify those that clustered with MYB sequences that are known to be involved in cell shape development. Direct application of dsRNA from the 3’ region of a selected floral specific cDNA to developing orchid flower buds resulted in altered flower epidermal cell shape. This report demonstrates the use of a simple approach using direct application of dsRNA to study the role of a transcription factor involved in regulation of flower development in orchid.

## Results

### Isolation and phylogenetic analysis of *R2R3 MYB* sequences from *D. hybrida*

**Fig. 1:**
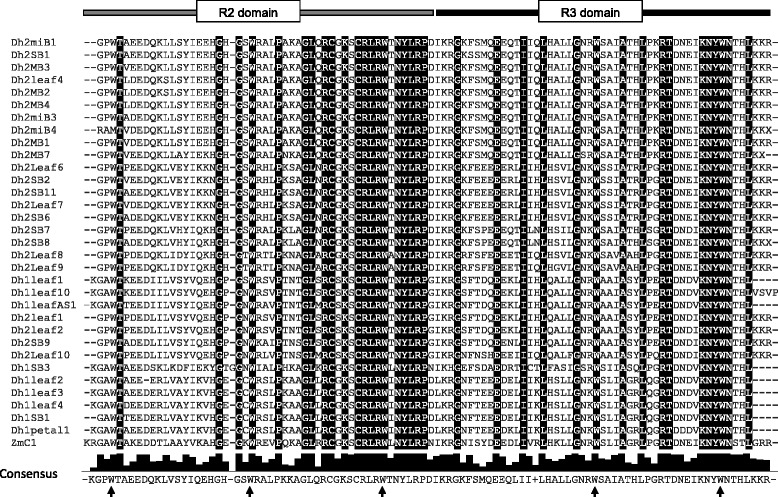
Alignment of putative amino acid sequences from 32 *D. hybrida* R2R3-MYB clones. Alignments were with DwMYB1: Dendrobium sp. XMW-2002-1 MYB 1 [GenBank: AAO49410]. The bar above the alignment indicates the R2 and R3 domains, underlined in grey and black, respectively. The majority consensus sequences are highlighted and the consensus of each amino acid is shown below the alignment. The region of R2R3 domains of *D. hybrida* sequences is defined according to DwMYB1: *Dendrobium sp*. XMW-2002-1 MYB 1 [GenBank: AO49410]. Arrows indicate the conserved regularly spaced three tryptophan residues in the R2 repeat and the two tryptophan and the phenylalanine residue in the R3 repeat

Thirty-two partial sequences (~300 bp) amplified from *D. hybrida* with degenerate MYB primers were identified to belong to the R2R3-MYB gene family based on the presence of conserved amino acids and two 50 amino acid imperfect repeat regions [27] (Fig. 1). The R2 repeat has three regularly spaced tryptophan residues and the R3 repeat includes two tryptophan and one phenylalanine residue (indicated by arrows in Fig. 1) as described by Stracke *et al.* [17]. Together the R2 and R3 domains encode three α-helices with the second and third helix forming a helix–turn–helix DNA binding domain.

**Fig. 2:**
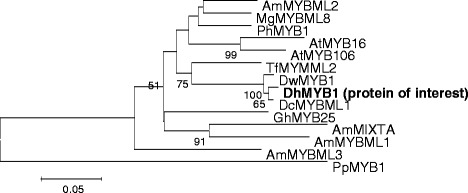
Phylogenetic relationship of amino acid sequences of DhMYB1 and R2R3-MYB proteins from subgroup 9. All amino acid sequences were for the full length of the coding sequences. AmMIXTA [GenBank: CAA55725.1], MIXTA-LIKE MYB gene AmMYBML1 [GenBank: CAB43399.1], AmMYBML2 [GenBank: AAV70655] and AmMYBML3 [GenBank: AAU13905] from *Antirrhinum majus*; AtMYB16 [GenBank: AED92146.1] and AtMYB106 [GenBank: AEE73615.1] from *Arabidopsis thaliana*; PhMYB1 [GenBank: CAA78386.1] from *Petunia hybrida*; DcMYBML1 [GenBank: ADD64500.1] from *Dendrobium crumenatum*, DwMYB1 [GenBank: AAO49410.1] from *Dendrobium sp*.; TfMYMML2 [GenBank: ACT78694] from *Thalictrum filamentosum*; MgMYBML8 [GenBank: ADV29952.1] from *Mimulus guttatus pop-variant IM62*; DhMYB1 from *Dendrobium hybrid* [GenBank: JX846911]. Bootstrap values are indicated at the nodes of the branches (values inferior to 50 % have been omitted). MYB 1 from *Pinus pinaster*, PpMYB1 [GenBank: ACA33839] was used as an outgroup to root the tree. The scale bar represents 0.05 substitutions per site

**Fig. 3:**
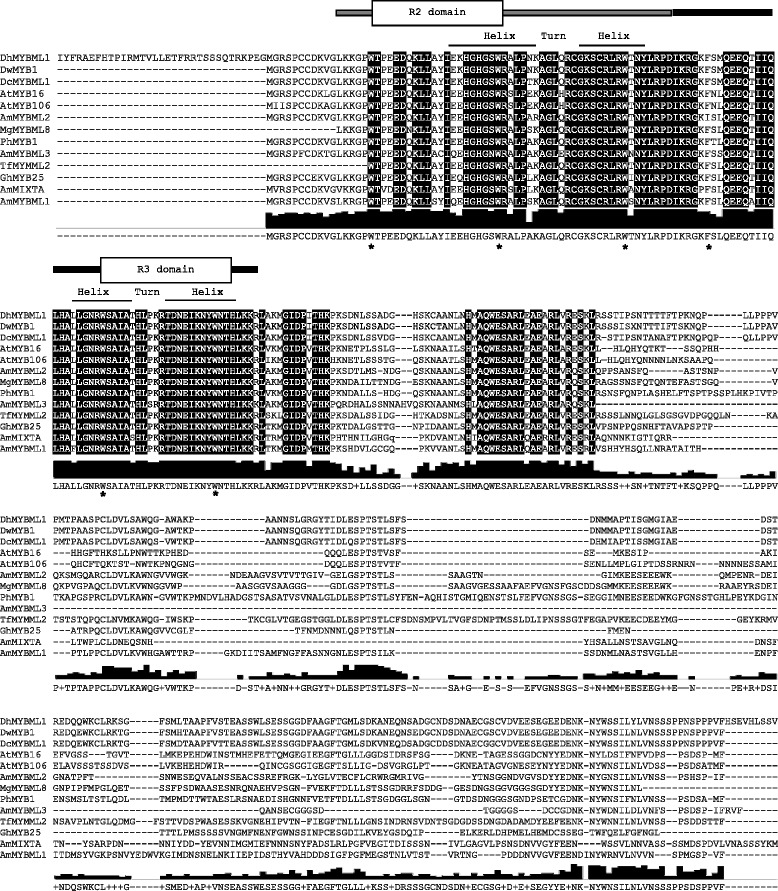
ClustalW amino acid sequence alignment of full length DhMYB1 with other subgroup 9 MYB sequences. The R2 and R3 domains are underlined in grey and black, respectively, whereas the positions of helix-turn-helix region are indicated by lines above the alignment. The majority consensus sequence is given at the bottom; conserved amino acids are highlighted and their conservation is shown as bars below the alignment. Dashes indicate gaps; asterisks indicate regularly spaced tryptophan residues. The R2R3 domains, helix-turn-helix regions and regularly spaced tryptophan residues are defined according to R1R2R3 type c-MYB protein [GenBank AAA48962]

Phylogenetic analysis of the 32 partial putative R2R3-MYB translated amino acid sequences from *D. hybrida* with MYB proteins from other plants, formed 22 subgroups with the MYB translated proteins from *D. hybrida* clustered into six subgroups. *i.e.,* subgroups 1, 4, 9, 10, 11 and 14 (Additional file 1). Subsequently, a full length cDNA (*DhMYB1*) was amplified from *D. hybrida* flower RNA using the partial R2R3 MYB sequence that is monophyletic with AmMIXTA and the translated full length sequence used for phylogenetic analysis, that grouped it together with DwMYB1 and DcMYBML1, whilst its sister group contained AmMIXTA and AmMYBML1 (Fig. 2). Full length cDNA sequence analysis revealed that *DhMYB1* cDNA shares 97 % identity with the *Dendrobium* sp. XMW-2002-1 MYB1 (*DwMYB1*) mRNA, complete cds and 73 % identity with the *Dendrobium crumenatum* MYBML1 mRNA, complete cds. Amino acid alignment of the DhMYB1 with other MYB sequences from subgroup 9, revealed high sequence similarity within the R2R3 domain at the N-terminus (amino acids 43 to 167) as shown in Fig. 3. Alignment of the translated full length DhMYB1 sequence together with those of MYBs in subgroup 9, showed high sequence similarity within the R2R3 domain at the N-terminus and the presence of the motif AQWESARxxAExRLxRES, previously described by Stracke *et al.* [17] within the C-terminal domain.

### Growth and development of *D. hybrida* floral buds

**Fig. 4:**
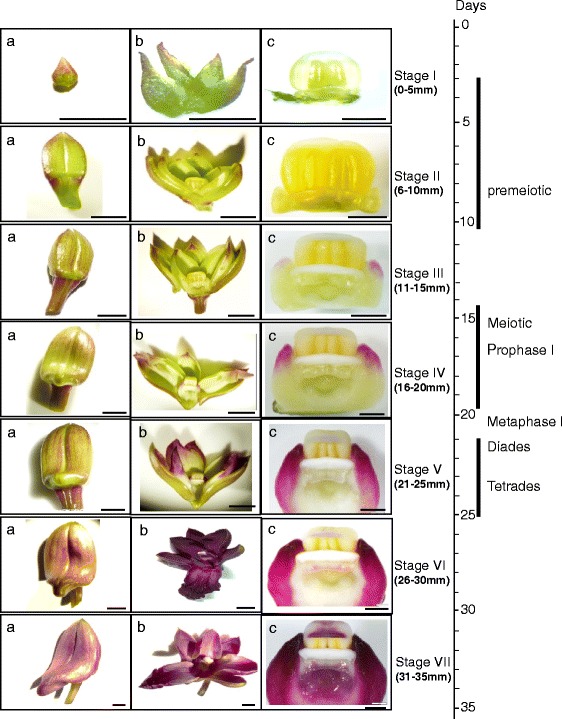
Flower bud developmental stages of *D. hybrida*. Stage 1 (0–5 mm), immature flower bud with light green surface; stage 2 (6–10 mm), small sized bud with light green surface; stage 3 (11–15 mm), medium-small sized bud with green adaxial surface and abaxial surface turning light purple; stage 4 (16–20 mm), medium sized bud with adaxial surface of perianth turning light purple; stage 5 (21–25 mm), medium- large bud with green adaxial surface and abaxial surface turning more purple and stage 6 (26–30 mm), large bud with purplish adaxial and abaxial surface; stage 7 (31–35 mm), mature bud with dark purple at adaxial and abaxial surface. **a** Bud morphology **b** Buds were opened and separated to show the developmental stage; **c** Column/anther morphology at different developmental stages. (**a** and **b**) bar = 50 mm, (**c**) bar = 1 mm

**Fig. 5:**
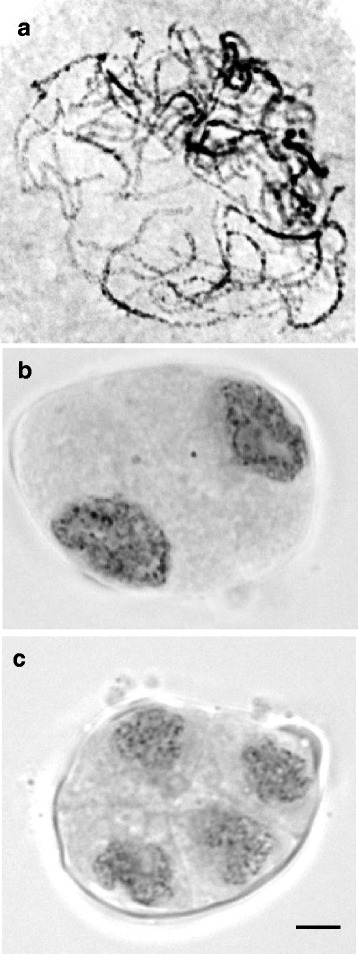
Male meiotic nuclei from floral buds of *D. hybrida.* **a** Meiotic prophase of a 16 mm bud at late zygotene to early pachytene showing thick, darker paired and thin, lighter unpaired chromosomes. **b** and **c** Meiotic cells from a 23 mm bud at the interphase between metaphase I and metaphase II (diade stage, **b**), and early tetrade stage with four equally sized meiotic products (**c**) Scale bar: 10 μm

Floral buds from *D. hybrida* were divided into seven developmental stages from emerged bud until mature bud, based on physical characteristics, in particular size as shown in Fig. 4. *D. hybrida* emerged buds typically took 6 days to reach 5 mm in length; 11 days to reach 10 mm in length; 15 days to reach 15 mm in length, 19 days to reach 20 mm in length; 22 days to reach 25 mm in length; 25 days to reach 30 mm in length; 29 days to reach 35 mm in length; 30 days to reach unfolding of petals and sepals and 32 days to reach anthesis. Pollinia from buds with different sizes were examined for meiotic stages. Floral buds from stages 1 and 2 showed premeiotic nuclei, while meiotic prophase at zygotene/early pachytene (Fig. 5a) were observed in a 16 mm buds (stage 4), and diades and tretades (Fig. 5b and c) were found in a 23 mm bud (stage 5). We therefore assume that meiosis starts during floral bud stage 3 and takes place during bud stages 4 to 5 (Fig. 4) and proceeds normally.

### Analysis of the expression levels of the *DhMYB1* cDNA in different floral bud stages

**Fig. 6:**
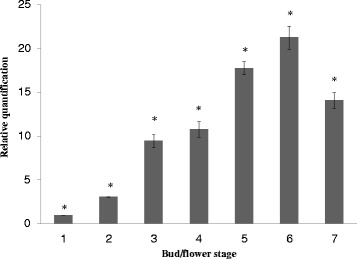
Expression levels of the *DhMYB1* cDNA in floral buds from stages 1 to7. The relative expression of *DhMYB1* cDNA was obtained by dividing the average number of *DhMYB1* transcript copies by the copy number of *D. hybrida β-actin* (endogenous control) for the same tissue. The lowest transcript level (*DhMYB1* cDNA in stage 1) was then set to a value of 1 and subsequently expression levels are reported relative to this number. Bars indicate standard error from amplification of 3 replicates of *DhMYB1* cDNA samples. Asterisks on the top of each bar highlight the differences in *DhMYB1* expression level according to a Tukey HSD comparison test (P < 0.05)

**Fig. 7:**
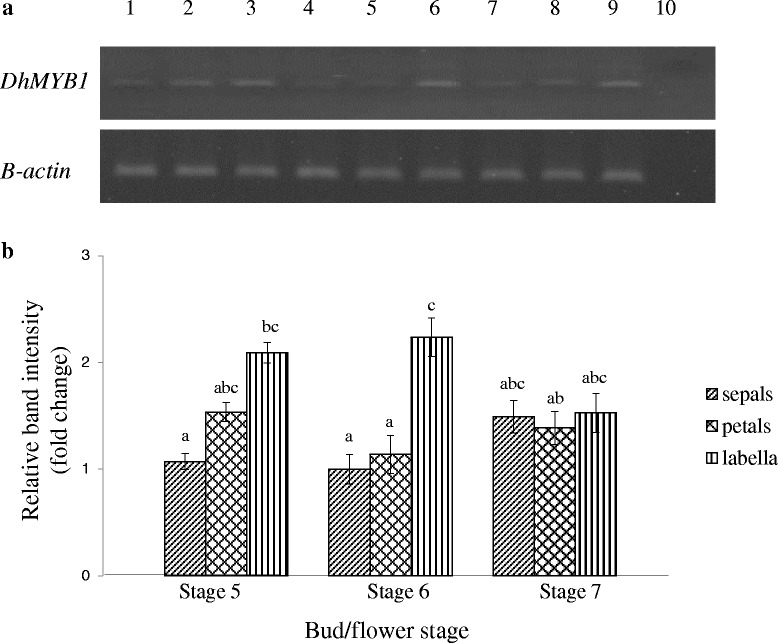
Relative expression levels of the *DhMYB1* cDNA in different floral tissues of floral buds. **a** Representative semi-quantitative RT-PCR results for *DhMYB1* and *β-actin* cDNA in sepals, petals and labella of stage 5 to 7 floral buds. Lane 1: stage 5 sepals; lane 2: stage 5 petals; lane 3: stage 5 labella; lane 4: stage 6 sepals; lane 5: stage 6 petals; lane 6: stage 6 labella; lane 7: stage 7 sepals; lane 8: stage 7 petals; lane 9: stage 7 labella; lane 10: negative control. **b** Relative intensity of PCR bands for *DhMYB1* normalized to the constitutive *β-actin* gene. Each bar represents the mean for three biological replicates with error bars indicating the SE. Letters indicate significant differences using one way ANOVA followed by Tukey HSD multi-comparison analysis test (P < 0.05)

To determine the pattern of *DhMYB1* gene expression, *DhMYB1* cDNA was quantified by real-time PCR for all floral bud stages (Fig. 6). The transcript level of *DhMYB1* increased gradually from stage 1 (0 – 5 mm bud length) to stage 6 (26 – 30 mm) and decreased at stage 7 (31–35 mm). One way ANOVA analysis and multi-comparison analysis (Tukey HSD test) on the average results of three independent experiments showed significant differences in *DhMYB1* expression from stage 1 to stage 7 (P < 0.05; Fig. 6). Following close examination of the plants treated with *DhMYB1* dsRNA and noting that only labellum cells showed altered phenotypes, the levels of *DhMYB1* cDNA in sepals, petals and labella of bud stages 5, 6 and 7 were determined by semi-quantitative RT-PCR. Expression of *DhMYB1* was generally higher in the labella compared to sepals and petals with the largest difference at stage 6 where expression was 2.2- and 2.0-fold higher than in sepals and petals, respectively (Fig. 7).

### Cell shape of the adaxial epidermis of the perianth of *D. hybrida*

**Fig. 8:**
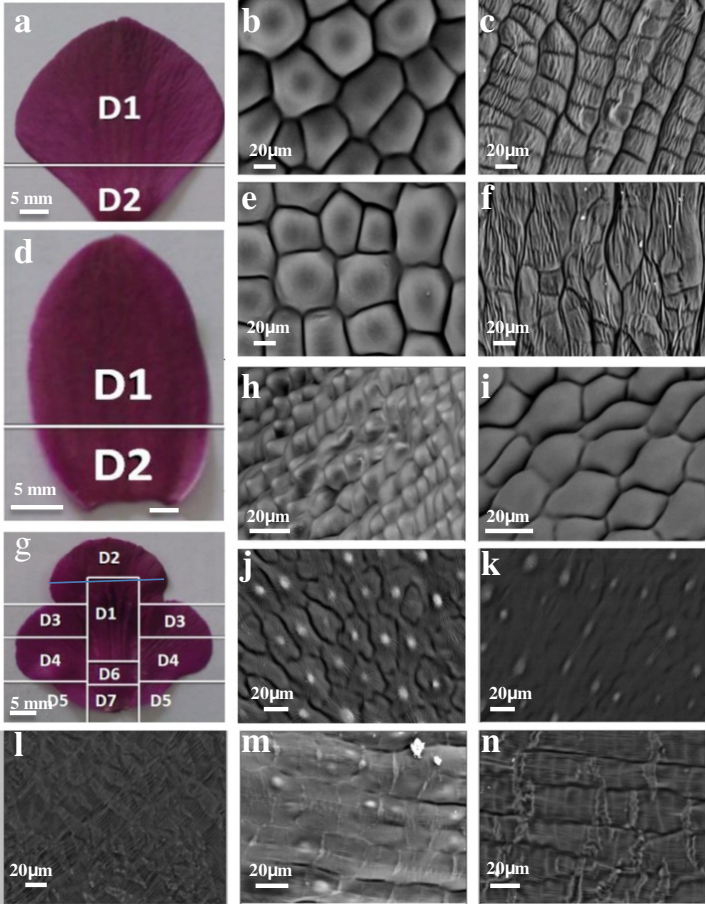
A diagram illustrating various shapes of adaxial epidermal cells in the perianth of *D. hybrida*. **a** Adaxial surface of petal divided to 2 domains. D1: domain 1; D2: domain 2. **b** Adaxial surface of petal domain 1- flattened epidermal cells **c** Adaxial surface of petal domain 2 - flattened epidermal cells with rectangular bases **d** Adaxial surface of sepal divided into 2 domains. D1: domain 1; D2: domain 2. **e** Adaxial surface of sepal domain 1 - flattened epidermal cells **f** Adaxial surface of sepal domain 2 - flattened epidermal cells with irregular bases **g** Adaxial surface of labellum divided to 7 domains. D1: domain 1; D2: domain 2; D3: domain 3; D4: domain 4; D5: domain 5: D6: domain 6; D7: domain 7. **h** Adaxial surface of labellum domain 1 - conical epidermal cells **i** Adaxial surface of labellum domain 2 - flattened epidermal cells. **j** Adaxial surface of labellum domain 3 - epidermal cells showed a single, central outgrowth of the outer, cuticularised wall. **k** Adaxial surface of labellum domain 4 - random cellular outgrowths. **l** Adaxial surface of labellum domain 5 - regular striations within the epidermal cells. **m** Adaxial surface of labellum domain 6 - flattened epidermal cells with rectangular bases, random conical cells on the surface. **n** Adaxial surface of labellum domain 7 - flattened epidermal cells with rectangular bases. Adaxial epidermis cell shapes were captured on fresh samples using variable pressure scanning electron microscopy with 400 × magnification

In order to characterize the shapes of floral adaxial epidermal cells, labella, petals and sepals of *D. hybrida* flowers at the anthesis stage were examined by scanning electron microscopy. The labellum in *D. hybrida* was more complex compared to the sepal and petal each of which was divided into two domains: the larger upper domain 1 with flattened epidermal cells and the smaller basal domain 2 with flattened rectangular or irregular epidermal cells (Fig. 8). In contrast, there were seven different types of epidermal cell shapes (which can be described as seven domains) on the adaxial surface of the labellum (Fig. 8).

### Analysis of expression of *DhMYB1* and phenotype of *D. hybrida* floral buds treated with *DhMYB1* dsRNA

**Fig. 9:**
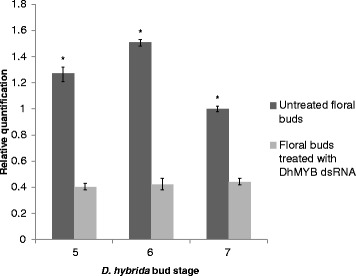
Gene expression in buds after treatment with dsRNA. Quantification of expression levels of the *DhMYB1* from untreated buds and buds treated with *DhMYB1* dsRNA at stages 5, 6 and 7. Relative expression of *DhMYB1* was obtained by dividing the average number of *DhMYB1* transcript copies by the copy number of *D. hybrida β-actin* (endogenous control) for the same tissue. The transcript level of *DhMYB1* RNA in stage 7 untreated floral buds was then set to a value of 1 and subsequently expression levels are relative to this number. Bars indicate standard error from triplicate amplification of the *DhMYB1* cDNA samples (n = 3). Transcript levels of *DhMYB1* gene in treated buds are significantly different from untreated control buds, P < 0.05

*D. hybrida* buds treated with *DhMYB1* dsRNA were compared to non-treated controls at stages 5, 6 and 7 (22 days post-treatment (dpt), 25 dpt and 29 dpt, respectively). While there was no obvious change in phenotype between treated and control flowers, there was a significant (P < 0.05) decrease in *DhMYB1* RNA levels of around 3.3-fold at stage 5, 4-fold at stage 6 and 2.4-fold at stage 7 (Fig. 9).

### Analysis of adaxial epidermal cell shape in the perianth of buds treated with *DhMYB1* dsRNA

**Fig. 10:**
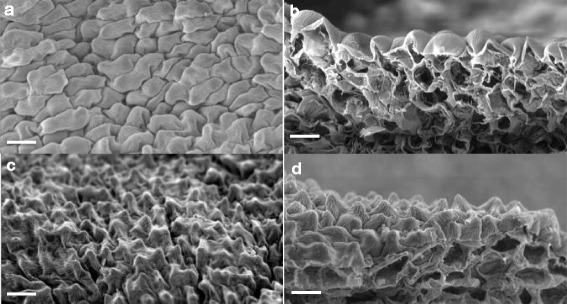
Comparison of adaxial epidermal cells from dsRNA treated and untreated perianth of *D. hybrida*. **a** SEM image of adaxial epidermis cell shape of domain 1 (D1) of flower labellum after treatment with *DhMYB1* dsRNA from *E. coli*. **b** cross section of a. **c** SEM image of adaxial epidermis cell shape of domain 1 (D1) of untreated control flower labellum*.* **d** cross section of c. Scale bar: 20 μm

Comparison of treated and control flower tissues by scanning electron microscopy showed no observable differences in adaxial epidermal cell shapes of sepals and petals, however, cells of domain 1 of the flower labella treated with *DhMYB1* dsRNA differed from those of the matching control. The treated flowers had relatively flattened epidermal cells whilst those of the control were conical (Fig. 10).

## Discussion

The MYB family of transcription factors are important regulators of biological development, represented by multiple genes within each eukaryotic species, including plants from unicellular algae to multicellular plants [15]. With an aim to study if direct application of dsRNA of a MYB gene family member can affect flower phenotype development, we first identified candidate R2R3 MYB gene sequences from *D. hybrida*, and also confirmed this to be a large gene family, with at least 32 distinct members. Although MYB subgoup 9 contains several genes of unknown function, for example *Dendrobium* sp. XMW-2002-1 *MYB1* and *D. crumenatum MYBML1* [27], this group was selected as the focus for functional study in *D. hybrida* as some MYB subgroup 9 family members have been associated with cell shape and pigment development including *AmMIXTA* [5] and *AmMYBML1* [21] and with regulation of cell shape in several plant species for example *PhMYB1* in *Petunia hybrida* [12] and *TfMYMML2* in *Thalictrum filamentosum* [22]. However, there are few reports of the expression profile of these genes at different flower developmental stages.

### Flower development and cell shape in *D. hybrida*

In order to characterise expression of the *DhMYB1* in this study, we first characterised stages of flower development, from immature bud to mature bud of *D. hybrida* into seven stages according to the flower bud length (Fig. 5) as has been similarly reported for *Dendrobium* X Jaquelyn Thomas ‘Uniwai Prince’ (UH503) [28] and *Dendrobium* Sonia ‘Earsakul’ [24]. In addition, we identified the time of meiotic division in *D. hybrida* as between late stage 3 to end of stage 5 (bud size in the range of 11 to 25 mm) with pollen formation estimated to occur at stage 6 (bud size in the range of 26 to 30 mm; Fig. 5). We also characterised adaxial epidermal cells of the flower perianth by SEM, finding that the petals and sepals each are divided into two distinct domains based on cell shape and size (Fig. 8b, c, e and f). A previous study with several species of orchid also described various cell types based on cell shape and size, but not in terms of domains within the petals, sepals or labella [29]. The presence of only flat epidermal cells in petals and sepals of *D. hybrida* (this study) is similar to that reported for *D.* Autumn Lace, *D.* Betty Goto, *D. canaliculatum* X *D. taurinum*, *D. gouldii* and *D. lasianthera* [29]. However, a recent study with *Phalaenopsis* OX Red Shoes [30] found that the adaxial epdidermal cells of the sepal were conical in shape and those on the labellum were flat, suggesting different species of orchids, such as *Dendrobium* and *Phalaenopsis*, may have different cell shape patterns. The labellum of *D. hybrida*, was the most complex part of the perianth, in terms of cell morphology, and was characterised into seven distinct domains (Fig. 8h-n). A conical cell shape was only observed in the labellum of *D. hybrida* and the cell shape in the petals and sepals of *D. hybrida* were apparently unchanged by reducing the expression of *DhMYB1*.

Conical epidermal cells are a common feature of petal tissues and have been reported to influence pollination by insects and birds *via* changes in surface texture and light reflection and hence, the perception of colour and surface area by pollinators [7, 10]. The presence of conical cells in the labellum, and not in the perianth of *D. hybrida*, thus correlates with a role in attracting pollinators to this part of the flower. In our study, the change in epidermal cell shape could not be perceived by the human naked eye and there was no apparent change in shape or color of the labella of treated compared to control flowers, even though these could be detected as reduction in *DhMYB1* mRNA and altered cell shape under the electron microscope. It is possible, however that the change in flower labellum cell shape, may be perceived by insects, as been reported in *Petunia* for flat-celled mutant lines that were less visited by bees compared to the conical-celled wild type flower [8]. Gene silencing using VIGS for other families of transcription factors, bHLH and MADS box, in the orchid *Phalaenopsis equestris* [9] and *PeSEP2* and *PeSEP3* in *Phalaenopsis* OX Red Shoes [30], resulted in changes in both cell and flower shape, while also not changing flower color . It was however reported that transient expression by particle bombardment of an R2R3 MYB transcription factor OgMYB1, from the orchid *Oncidium Gower Ramsey*, induced the formation of red pigments in the flower labellum [31]. In the latter study, the normal expression pattern was found only in petals and sepals, but the rest of the pigment pathway remained active so could be activated when the MYB was ectopically expressed in the labellum. Thus, not unexpectedly, it appears that several families of transcription factor must work together to result in the shape and colour of floral cells and organs and that changes that are not visible to humans may still have a biological and ecological impact. In addition to its role in attraction of insect pollinators, the unique and special nature of the orchid labellum is an important commercial quality of these plants and the characterization of the different regions, as in the current study, if extended to other species and cultivars of orchid, could be a useful morphometric marker for molecular breeding, similar to the characterization of petal epidermal cells of legume flowers which has been used as a micromorphological marker of different petal identity [32, 33].

### *DhMYB1* is expressed during flower development

In this study we focused on a candidate from the MYB9 subgroup and observed that *DhMYB1* expression from immature bud through to mature bud, is consistent with a role in flower development (Fig. 6). The maximum observed expression of *DhMYB1* in the stage 6 floral bud, coincides with the fully formed flower at around 6 to 7 days before opening (Fig. 4). At this stage, further flower development mainly consists of cell expansion and flower opening, thus the expression of *DhMYB1* may relate to a role in the continued regulation of cell shape during cell expansion as the flower matures. Members of the MYB9 subgroup have been reported to have various roles in flower development, and here we observed that the direct application of dsRNA sharing sequence identity with *DhMYB1*, significantly reduced the level of *DhMYB1* RNA (Fig. 9) and resulted in altered cell shape in the orchid flower labellum (Fig. 10). The change from (wild type) conical shaped cells to flattened cells, is very similar to that reported in the flowers of *mixta* mutants in *Anthirrhinum majus* [5, 34] and *phmyb1* mutants in *Petunia hybrida* [12], supporting orthologous roles for *DhMYB1, PhMYB1* and *AmMIXTA*. The expression pattern of *DhMYB1* in *D. hybrida* is comparable with that reported for *PhMYB1*in petunia flowers [12] where the transcript level of the *DhMYB1* and *PhMYB1* increases gradually with the increasing size of floral bud and peaks when the flower bud is around 70 % of its maximum size. In contrast, the expression patterns reported for *AmMYBML2* and *AmMIXTA* in *Anthirrhinum majus* differ, with the former expressed relatively late, peaking when the corollas of the flowers are just opened and the expression of the latter peaking slightly earlier when the flowers are around half their final size. Interestingly, the phylogeny for this group (Fig. 2) also places the PhMYB1 and DhMYB1 slightly closer together. The effect of dsRNA (and reduced mRNA) on flower cell shape, was only observed in the labella and not the petals or sepals of *D. hybrida* (Fig. 10), thus we suggest that the function of *DhMYB1* relates to normal cell development of conical shaped cells throughout the maturity of the flower, and which we observed were confined to the labella of *D. hybrida*, but are found in the petals in both *Petunia* and *Antirrhinum*. This is also supported by the expression pattern of *DhMYB1* which was higher in the labella compared to the petals and sepals, which lack conical shaped epidermal cells (Fig. 7).

### Direct application of dsRNA is a useful approach to study flower developmental gene function

Overexpression and RNA interference (RNAi) technology have proven to be useful tools to study the role of MYB family proteins during floral development and to improve flower quality and quantity [35]. However, in slow growing species such as orchids, stable transformation methods are not practical and thus VIGS and other transient expression and gene silencing approaches are more time and cost effective. VIGS has been demonstrated as an effective method for gene silencing of MADS box transcription factors in *Phalaenopsis* orchids [9] and to have up to 95.8 % silencing efficiency [36]. A recent study in *Dendrobium* Sonia [24] demonstrated the utility of an agroinfiltration RNAi approach to assay gene function in floral tissue of orchids. In our study, RNAi knock down of the expression of *DhMYB1* using *DhMYB1* dsRNA was able to produce a clear loss of function phenotype (altered epidermal cell shape) far more rapidly than could have been achieved *via* transgenic approaches, thus this may have potential for use in biotechnological applications as well as for research. This approach has been used successfully in animal systems such as *Caenorhabditis elegans* [37] but in plants previous reports are limited to use for viral resistance, for example in tobacco [38], maize [39] and orchid [26]. In the current study, this approach was found to reduce transcript levels of *DhMYB1* of floral buds in *D. hybrida* successfully. Moreover, mechanical inoculation of RNA using bacterially expressed genes is a protocol that is simple and quick when compared with using either the *in vitro* synthesis of large amounts of RNA or the introduction of a transgene into plants. We envision that for some gene candidates, altered phenotypes would also be visible by naked eye, for instance, loss or reduction of pigmentation or changes in organ identity.

## Conclusions

The development of *Dendrobium hybrida* flower buds was characterised into seven stages with meiosis occurring between stages 3 to 5 (bud size in the range of 11 to 25 mm) and pollen formation estimated to occur at stage 6 (bud size in the range of 26 to 30 mm). We also characterised adaxial epidermal cells of the flower perianth by SEM, finding that the petals and sepals each are divided into two distinct domains based on cell shape and size, while the labella had seven domains. We identified and cloned a full length *R2R3-MYB* cDNA sequence, *DhMYB1,* othologous to MYB sequences that are known to be involved in cell shape development and using *DhMYB1* gene sequences, demonstrated that the direct application of dsRNA to developing flower buds can reduce levels of endogenous transcripts in flowers. This leads us to suggest a role for *DhMYB1* in regulating the conical shape of the labella epidermal cells throughout flower development. This method is relatively rapid and inexpensive compared to vector based genetic transformation. We suggest that direct application of dsRNA to flowers can be an alternative method for cell transformation that may be useful for horticulture, in particular with respect to flower modification.

## Methods

### Plant materials

Hybrid plants (*Dendrobium hybrida*) clonally propagated from an unregistered cross of *Dendrobium* bobby messina and *Dendrobium* chao phraya were used for all experiments. Plants were purchased from Cheah Wah Sang Orchid Farm in Shah Alam, Selangor, Malaysia and grown in pots with charcoal as a root medium.

### PCR amplification of the MYB domains, cloning and sequence analysis

Total RNA was extracted from floral and young leaf tissues with a CTAB method [40]. First-strand cDNA synthesis was carried out by using High capacity cDNA reverse transcription kit (Applied Biosystem, USA) according to the manufacturer’s instructions. A 2 μl portion of first-strand cDNA reaction was used in i-Taq™ DNA polymerase PCR kit with two sets of degenerate primers (Dh1-MYB F: AAAGGTGCWTGGACYRMKGAAGAAGA and Dh1-MYB R: AGRTGRGTGTTCCARTARTTYTTGACRTC; Dh2-MYB F: AGGGCCATGGACWSYAGAMGAAGAYC and Dh2-MYB R: GAACTAYTGGAACACWCATYTAAAGAARCG) (custom synthesised by Intron Biotechnology, Korea). PCR reactions were cycled 30 times for 45 s at 94 °C, 45 s at 58 °C, and 1 min at 72 °C, respectively. RT-PCR products of approximately 300 bp were separated on 1 % agarose gel, extracted and cloned into pGEM®-T Easy Vector (Promega, USA). The DNA sequence of the cloned inserts was determined by Sanger sequencing using a commercial service (First BASE Laboratories Sdn Bhd, Malaysia).

### 3’ and 5’ Rapid amplification of cDNA ends – polymerase chain reaction (5’ and 3’ RACE-PCR)

Full length sequence of a *DhMYB1* cDNA was obtained by 3’ and 5’ RACE using GeneRacer™ Kit (Invitrogen™, USA) according to the manufacturer’s instructions. The 5’ RACE-PCR was amplified using the GeneRacer™ 5′ Primer (CGACTGGAGCACGAGGACACTGA) and DhMYB 3’ primer (GCTTGCATACATTGAGAAGCATGGGC) whereas 3’ RACE-PCR was amplified using a DhMYB 5’ primer (AGACCACCTGTTGCCAAGGA) and GeneRacer™ 3′ Primer (GCTGTCAACGATACGCTACGTAACG). The PCR product was cloned by using Zero Blunt® TOPO® PCR Cloning Kit (Invitrogen™) and sequenced.

### Phylogenetic analyses

Thirty-two isolated *D. hybrida* cDNA sequences were translated into putative partial amino acid sequences using EMBOSS Transeq (http://www.ebi.ac.uk/Tools/st/emboss_transeq). The 32 partial amino acid sequences were aligned with DwMYB1 [GenBank: AF485892.1] [27] using EMBL-EBI ClustalW2 (http://www.ebi.ac.uk/Tools/msa/clustalw2/). Phylogenetic analysis was performed using IT3F: An Interspecies Transcription Factor Function Finder for Plants (http://jicbio.bbsrc.ac.uk/IT3F) with the 32 isolated putative partial protein sequences and other plant MYB superfamily R2R3 members from the GenBank database (the accession numbers and details are listed in Additional file 2). One candidate gene from subgroup 9 of the clustered R2R3 MYB gene sequences (subsequently named *DhMYB1*) was selected and the full-length cDNA amplified for functional study. The phylogenetic relationship of the amino acid sequence of DhMYB1 and other MYB proteins from subgroup 9, AmMIXTA [GenBank: CAA55725.1], MIXTA-LIKE MYB gene AmMYBML1 [GenBank: CAB43399.1], AmMYBML2 [GenBank: AAV70655] and AmMYBML3 [GenBank: AAU13905] from *Antirrhinum majus*; AtMYB16 [GenBank: AED92146.1] and AtMYB106 [GenBank: AEE73615.1] from *Arabidopsis thaliana*; PhMYB1 [GenBank: CAA78386.1] from *Petunia hybrida*; DcMYBML1 [GenBank: ADD64500.1] from *Dendrobium crumenatum*, DwMYB1 [GenBank: AAO49410.1] from *Dendrobium* sp.; TfMYMML2 [GenBank: ACT78694] from *Thalictrum filamentosum*; MgMYBML8 [GenBank: ADV29952.1] from *Mimulus guttatus pop-variant IM62* was carried out using Molecular Evolutionary Genetics Analysis (MEGA 4) (Center for Evolutionary Medicine and Informatics, Tempe, USA). PpMYB1 [GenBank: ACA33839] was used as an outgroup to root the tree and it was generated by using neighbour-joining analysis.

### Plasmid construction

Bacterial expression vector pL4440/*DhMYB1* was constructed with the potential to express double-stranded RNA corresponding to partial C-terminal sequences of *DhMYB1* under the control of a double T7 RNA polymerase promoter. The construct contained 430 bp (nts 769 to 1198) of *DhMYB1* [GenBank: JX846911], amplified by RT-PCR using RNA from *D. hybrida* young floral bud as a template. To generate the pL4440/*DhMYB1* construct, primer 1, (GCGGCCGCCCTAGGAAGAGGATACACCATTGAC; *Not* I sequence (underlined) plus nts 769 – 794 nt of the *DhMYB1* gene sequence) and primer 2 (CCCATGGGGTGGCGGTGAATTTGGA); *Nco* I sequence (underlined) plus nts 1198 to 1181 of the *DhMYB1* gene sequence) were used to amplify the *DhMYB1* gene fragment which was gel purified and digested with *Not* I and *Nco* I, then ligated between the *Not* I and *Nco* I cloning sites in pL4440. The plasmid was introduced into the RNase III-deficient *E. coli* strain HT115 (DE3) [37] using a standard CaCl_2_ transformation protocol [41].

### Production of crude bacterial lysates containing *DhMYB1* dsRNA

A clone of the RNase III-deficient *E. coli* strain HT115 (DE3) harbouring the vector pL4440/DhMYB was maintained on solid LB medium supplemented with 100 μg/ml ampicillin and 12.5 μg/ml tetracycline. Preparation of *DhMYB1* dsRNA was by induction of this clone in liquid medium based on the method of Timmons *et al.* [37] as described in Lau *et al.* [26] but omitting the RNA annealing step.

### Treatment of orchid plants

For each experimental replicate, crude bacterial extract containing *DhMYB1* dsRNA was inoculated onto three orchid plants for each treatment and control groups with flowers at bud length 0–5 mm (stage 1). Mechanical inoculation was carried out by gently rubbing 50 μl containing 2 μg/μl of crude bacterial extract onto the flower bud at 5 day intervals using a latex-gloved finger. The plants were left for 30 min before rinsing off any debris under slow running tap water. The plants then were grown at 25 °C with a 12 h photoperiod. Treated and untreated buds were harvested at bud stages 5, 6 and 7, *i.e.,* 22 days post-treatment (dpt), 25 dpt and 29 dpt, respectively. Total RNA was extracted and expression levels of *DhMYB1* were analysed by RT-qPCR in three independent experiments (n = 3 per experiment). Images of floral buds were captured prior to RNA extraction.

### Analysis of *DhMYB1* gene expression

Samples of total RNA isolated using a CTAB method [40] were treated with DNase I (Invitrogen™). Two microgram of each RNA sample was used to prepare cDNA with High Capacity cDNA Reverse Transcription Kits (Applied Biosystems, USA) using random hexamers. For quantitative PCR experiments, *DhMYB1* RNA was quantified by two-step reverse transcription-quantitative real-time polymerase chain reaction (RT-qPCR) using the *DhMYB1* specific primers (DhMYB F: TGCTGTCGGATAAAGCCAATG and DhMYB R: GGTGGCGGTGAATTTGGA) and *β*-actin gene primers (*β*-actin F: TGGGCACCTAAATCTCTCAGC and *β*-actin R: GTCAGGGACATCAAGGAGAAG) in a 20-μL PCR mixture containing 2 μL of cDNA, 0.5 mM of each primer and Power SYBR® Green PCR Master Mix kit (Applied Biosystems, USA). Amplification conditions used an initial DNA Polymerase activation at 95 °C, 10 min, followed by 40 cycles of amplification with denaturing at 95 °C for 15 s, annealing and extension at 60 °C for 1 min using a 7500 Real Time PCR System (Applied Biosystems). SDS 1.3.1 (Sequence Detection Software) was used to create a relative quantification (ddCt) plate and the dissociation curves. *β*-actin was used as the endogenous reference for the normalization of the expression levels of the target CP gene [42] and the non-treated control sample was used as the calibrator. The relative quantification minimum (RQmin) ratio relative quantification maximum (RQmax) limit was set at 95 % confidence.

Semi-quantitative PCR was performed using a 20 μL PCR mixture containing 2 μL of cDNA, 5 pmoles of each primer, 1X PCR buffer, 0.25 mM of each dNTP and 1U of *i-Taq* DNA polymerase (iNtRON Biotechnology, Inc., Korea). Initial denaturing was set at 94 °C, 2 min, followed by 25 cycles of amplification with denaturing at 94 °C for 20 s, annealing at 60 °C for 10 s and extension at 72 °C for 30 s, and a final extension at 72 °C for 5 min. The amplified products were separated on 1.5 % (w/v) agarose gel, followed by image capture and band intensity analysis using AlphaImager HP (AlphaInnotech, USA) with AlphaInotech – Alphaview analysis software. *β-actin* was used as reference gene for relative quantification (using the primers as mentioned above).

### Statistical analysis

Relative quantification of *DhMYB1* cDNA at different bud stages (ranging from 0–35 mm) and different floral organs (sepals, petals and labella) were analysed by ANOVA one tailed test whereas relative quantification of *DhMYB1* cDNA between untreated control and treated bud stages 5, 6 and 7 (21–35 mm) were analysed by ANOVA two tailed test using statistica version 10 for Windows (StatSoft, USA). Multiple comparisons were performed with the Tukey HSD test. P < 0.05 was considered to be significant.

### Cytology

Flower buds of different stages were fixed in 100 % ethanol : glacial acetic acid 3:1 at room temperature for 1 h and then transferred to 70 % ethanol for storage at −20 °C. Buds were dissected under a stereo-microscope and pollinia were placed in a drop of acetocarmine on a microscope slide. Meiocytes were removed, squashed under a cover slip and analysed for meiotic stages by through-light microscopy. Images were taken on a Nikon Eclipse i80 microscope using a cooled CCD camera and NIS-Elements 4.0 imaging package.

### Scanning electron microscopy

*D. hybrida* labellum, petals and sepals were cut into small pieces and fixed in 2 % (w/v) glutaraldehyde in distilled water at 4 °C for 16 h, followed by 2 % (v/v) aqueous osmium tetroxide in distilled water at 4 °C for 16 h. Then, the samples were washed with distilled water two times for 15 min and dehydrated through a standard ethanol series. Next, samples were infiltrated through ethanol and acetone mixtures (3:1, 1:1 and 1:3 of ethanol: acetone) for 15 min each. After that, the samples were incubated in acetone for 1 h, and then dehydrated samples were subjected to critical point drying at 31.5 °C, 1100 psi using a CPD 7501 instrument (Poloran, UK). Dried samples were mounted on specimen stubs and dried for 16 h in vacuum desiccators then were coated with gold and examined using a JSM 6400 scanning electron microscope (SEM) (Jeol Ltd., Japan). Cell shapes were analysed from SEM micrographs at 100-, 400- and 500-times magnification. Fresh *D. hybrida* labella, petals and sepals were mounted on specimen stubs and examined using a Leo 1455 VP-SEM (Angstrom Scientific Inc., US) and at 400-times magnification.

### Availability of supporting data

Phylogenetic relationships and subgroup designations in MYB proteins from *D. hybrida* (*Dh*) and the list of the R2R3 MYB proteins are included in Additional files 1 and 2.

## Additional files

Additional file 1:
**Phylogenetic relationships and subgroup designations in MYB proteins from** ***D. hybrida*** **(** ***Dh*** **).** Phylogram made using Interspecies Transcription Factor Function Finder (IT3F) *At: Arabidopsis* thaliana; *Os*: *Oryza sativa*, *Am*: *Antirrhinum majus*, *Gh*: *Gossypium hirsutum*, *Fa*: *Fragaria ananassa*, *Eg*: *Eucalyptus gunnii* and *Le*: *Lentinula edodes*. Black: *Arabidopsis*; Yellow: monocots; Green: legumes; Red: *D. hybrida* from this study; Blue: MYB genes with known function; Red dot: gene duplication; Blue dot: Genes in tandem. (ZIP 339 kb) Additional file 2:
**Table S1.** Details for the R2R3 MYB proteins used in the Additional file 1. (DOCX 40 kb)
