# Supplementary material for: dsRNA silencing of an R2R3-MYB transcription factor affects flower cell shape in a Dendrobium hybrid
Source: BMC Plant Biol. 2015 Aug 11;15:194. doi: 10.1186/s12870-015-0577-3 (PMC4542095; doi:10.1186/s12870-015-0577-3)
Supplement: Additional file 2: — Table S1. Details for the R2R3 MYB proteins used in the Additional file 1. (DOCX 40 kb) [file 12870_2015_577_MOESM2_ESM.docx]

Subgroup 14


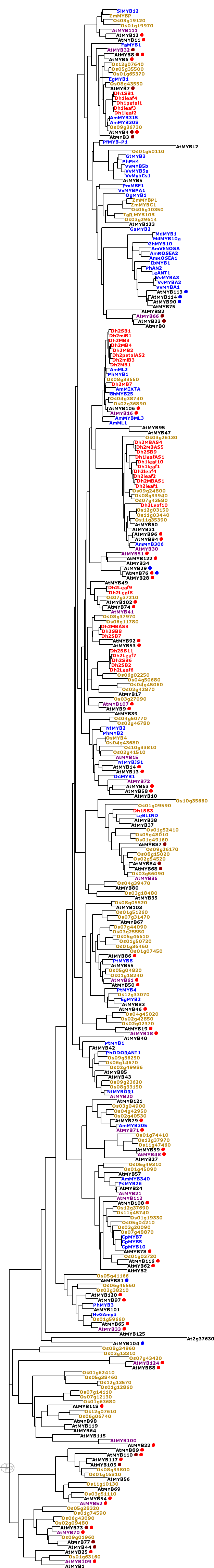


Subgroup 13

Subgroup 16

Subgroup 19

Subgroup 20

Subgroup 18

Subgroup 25

Subgroup 21

Subgroup 22

Subgroup 23
